# 

**Published:** 1905-11-15

**Authors:** 


### CONTENTS.

Event and Comment - - - - - - - - - 541

Cocain Pressure Anesthesia—Its Dangers and Failures, By E. T. Loeffler, B.S., D.D.S. 546

Importance of Occlusion in Crown and Bridge-work, By R. B. Howell, D.D.S. 554

Tests on the Inlay Cement Problem, By Joseph Head, M.D., D.D.S. 564

The Life of an Army Dentist, - By Samuel W. Hussey, D.D.S. 576

The Antiseptic Value of Chloretone, - By T. A. Gormly, D.D.S. 580

Indents - - - - - - - - - - - - - 581

Announcements - - - - - - - - - - 591

